# Identifying key mutations of radioresponsive genes in esophageal squamous cell carcinoma

**DOI:** 10.3389/fimmu.2022.1001173

**Published:** 2022-09-02

**Authors:** Xin Xu, Yuming Wang, Yongrui Bai, Jun Lu, Yuntao Guo, Xiaohang Wang, Ling Rong, Jianmin Tang, Xiumei Ma, Jun Ma, Lei Zhang

**Affiliations:** ^1^ Department of Radiation Oncology, Renji Hospital, School of Medicine, Shanghai Jiao Tong University, Shanghai, China; ^2^ Department of Thoracic Surgery, Renji Hospital, School of Medicine, Shanghai Jiao Tong University, Shanghai, China; ^3^ Department of Research, Medical Laboratory of Nantong Zhongke, Nantong, China; ^4^ Department of Bioinformatics, Medical Laboratory of Nantong Zhongke, Nantong, China; ^5^ Eye Institute, Eye & Ear, Nose, and Throat Hospital, Shanghai Medical College, Fudan University, Shanghai, China

**Keywords:** ESCC, radiotherapy, NOTCH1, survival, immune

## Abstract

**Background:**

Radiotherapy plays an important effect on the standard therapy of esophageal squamous cell carcinoma (ESCC). However, the efficacy of the therapy is limited and a few patients do not achieve satisfactory treatment results due to the existence of radiation resistance. Therefore, it is necessary to identify the potential predictive biomarkers and treatment targets for ESCC.

**Methods:**

We performed the whole-exome sequencing to determine the germline and somatic mutations in ESCC. Functional enrichment and pathway-based protein-protein interaction analyses were used to ascertain potential regulatory networks. Cell survival and cell death after treatment with radiotherapy were determined by CCK-8 and LDH release assays in ESCC cells. The correlations of NOTCH1 and tumor immune infiltration were also analyzed in ESCC.

**Results:**

Our results showed that 344 somatic and 65 germline differentially mutated genes were detected to be radiosensitivity-related loci. The tumor mutational burdens (TMB) or microsatellite instability (MSI) were not significantly correlated with the response to radiotherapy in ESCC patients. Pathway-based protein-protein interaction analyses implied several hub genes with most nodes (such as PIK3CA, NOTCH1, STAT3 and KDR). The *in vitro* studies showed that the knockdown of NOTCH1 inhibited cell survival and rendered more cell death after the treatment with radiotherapy in ESCC cells, while NOTCH1 overexpression had the opposite effects. Moreover, NOTCH1, frequently up-regulated in ESCC, was negatively correlated with activated B cell and immature dendritic cell in ESCC. High expression of NOTCH1 was accompanied with the low levels of some immunotherapy-related cells, including CD8(+) T cells and NK cells.

**Conclusions:**

These results indicate the differences of the germline mutations and somatic mutations between the radiosensitive and radioresistence groups in ESCC and imply that NOTCH1 plays important roles in regulating the radiosensitivity of ESCC. The findings might provide the biomarkers and potential treatment targets for improving the sensitivity to radiotherapy in ESCC.

## Introduction

Esophageal carcinoma (EsC), which is characterized by poor prognosis, high mortality rate and distinct epidemiologic pattern, is one of the most prevalent malignant tumors in the world. According to the statistical data from World Cancer Research Fund in 2018, EsC is the seventh most common cancer in men and the 13th in women (1). There are two main types of EsC. Esophageal adenocarcinoma is developed at the junction of the esophagus and stomach. Esophageal squamous cell carcinoma (ESCC), which occurs in the upper part of the esophagus, is the major subtype and accounts for the vast majority of cases (2). ESCC usually remains asymptomatic until extensive local, regional, or distant spread has occurred and ranks the sixth leading cause of cancer-related death (1). Surgical resection combined with the neoadjuvant chemoradiotherapy is considered as the standard treatment for ESCC. However, some patients have to only receive radical chemoradiotherapy because they do not meet the surgical indication. Although the chance of cure with radio-therapy is quite low, a significant portion of patients will receive palliation (3). Therefore, radiotherapy plays an important effect on the comprehensive treatment for ESCC.

Resistance is still considered as the major cause of radiation treatment failure for ESCC patients (4). Due to the existence of inherent or acquired radiation resistance, some patients failed to achieve enormous therapeutic effects, resulting in the metastasis or high recurrent rate, and ultimately death. Therefore, it is necessary to identify the critical factors involved in regulating the sensitivity of radiotherapy for ESCC, which will help to improve its efficiency. It is reported that the noncoding RNA NORAD induced by radiation facilitates radiotherapy resistance *via* the EEPD1/ATR/Chk1 pathway in ESCC (5). The sensitivity to concurrent chemoradiotherapy was increased by STAT3β by promoting cellular necroptosis in ESCC (6). Inhibition of carbonic anhydrase IX alters hypoxic tumor micro-environment and increases the efficacy of radiotherapy in ESCC (7). Nevertheless, the molecular mechanisms of radioresistance in ESCC have not been fully elucidated and still need to be further determined.

In the present study, whole-exome sequencing was utilized to identify mutations predicting benefits from radiation therapy in ESCC patients. We examined the germline and somatic mutations in ESCC and found several critical mutations of candidate genes potentially associated with the response to radiotherapy. Our study also showed that NOTCH1, which was mutated in the radiosensitive group, negatively regulated the response to radiotherapy in ESCC cells. The results provide some potential predictive biomarkers and therapy targets for improving the efficiency of radiotherapy in ESCC.

## Materials and methods

### Tissue and blood samples

We collected the formalin-fixed paraffin-embedded tumor tissues and their paired normal blood DNA from six Chinese ESCC patients. Tumor cell purity was assessed in hematoxylin and eosin (H&E) sections. At least 5 slices of 10 μm of thickness were cut from the paraffin block and tumor regions were scraped according to the assessment of tumor enriched area. For these six patients, all of them had received radical surgery to make sure none tumor tissues left pathologically. After surgery, all patients were received intensity-modulated radiation therapy (IMRT). The clinicopathologic characteristics of ESCC patients were provided in **Table 1**. According to the responses to radiotherapy, the ESCC patients were divided into the sensitive group (group S) and radioresistant group (group NS). Group NS indicated that no response to radiotherapy was achieved in ESCC patients, while the sensitive group (group S) meant that complete response to radiotherapy was achieved. All patients agreed and signed informed consent before recruitment to the study, and the ethical committee of Ren Ji Hospital, Shanghai Jiao Tong University School of Medicine has approved the studies.

**Table 1:** Clinicopathologic characteristics of ESCC patients who received radiotherapy.

	NS1	NS2	NS3	S1	S2	S3
**Age**	57	77	57	60	67	52
**Gender**	Male	Male	Male	Male	Male	Male
**Histology**	ESCC	ESCC	ESCC	ESCC	ESCC	ESCC
**TNM**	IIIA	IIIA	IIB	IIIA	IIIA	IIB
**Dose (Gy)**	50	50	50	50	50	46
**Radio-sensitive**	No	No	No	Yes	Yes	Yes
**Relapse**	Yes	Yes	Yes	No	No	No
**Overall Survival (months)**	13.83	27.00	15.77	51.20	68.20	47.40
**Status**	Die	Die	Die	survive	survive	survive

### Sequencing analysis

Whole-exome sequencing was performed by Precisiongenes Technology, Inc. following a protocol including genomic DNA extraction, DNA library construction, exome capture by SureSelect Clinical Research Exome V2 Capture Kits (Agilent, SantaClara, California, United States) and paired-end 150bp sequencing on Illumina NovaSeq 6000 (Illumina Inc). Tumor and normal library pairs were sequenced on a single flow cell. And germline-only samples were run on the other flow cell. Whole length of probe Clinical Research Exome V2 is 67.3Mb.

Raw fastq data achieved from Illumina were firstly checked quality control, removed adapters and low-quality reads with FASTP (8). Secondly, Burrows Wheller aligner (BWA) MEM algorithm was applied to align high-quality clean data onto the hg19 reference genome (GCA_000001405.1) with default options. Thirdly, Samtools was used to convert the SAM file into BAM (9). Fourthly, Picard Toolkit (https://github.com/broadinstitute/picard) was carried out to sort mapped reads according chromosome coordinate, mark PCR duplicates and fix paired-end information in BAM files.

For germline mutations, single-nucleotide polymorphisms (SNPs), small insertions and deletions (INDELs) were discovered and filtered following the Genome Analysis Toolkit (GATK, https://software.broadinstitute.org/gatk/) recommendations of DNAseq best practice guidelines (https://gatk.broadinstitute.org/hc/en-us/articles/360035535932-Germline-short-variant-discovery-SNPs-Indels-) (10).

For tumor somatic mutations, candidate single-nucleotide variants (SNVs) and INDELs were called by Mutect2. Then, an estimate of the fraction of reads due to cross-sample contamination for each tumor sample and an estimate of the allelic copy number segmentation of each tumor sample were emitted by Get Pileup Summaries and Calculate Contamination. Finally, somatic mutations were filtered by Filter Mutect Calls. Notably, Mutect2, Get Pileup Summaries, Calculate Contamination and Filter Mutect Calls are components of GATK. And the process mentioned above was referred to website (https://gatk.broadinstitute.org/hc/en-us/articles/360035894731-Somatic-short-variant-discovery-SNVs-Indels-).

### Identification of differentially mutated genes

65 germline differentially mutated genes and 344 somatic differentially mutated genes were filtered from all mutation genes following the criteria that any one of the mutated genes was not in both the radiation-resistant group and the radiation-sensitive group at the same time.

### Tumor mutation burden and microsatellite instability

Tumor mutation burden (TMB) is defined as the number of somatic SNVs, and INDELs per megabase of genome examined (mut/Mb) (Analysis of 100,000 human cancer genomes reveals the landscape of tumor mutational burden). All SNVs and INDELs in the captured region of targeted genes, including synonymous mutations, are initially counted before filtering as described below. Synonymous mutations are counted in order to reduce sampling noise. While synonymous mutations are not likely to be directly involved in creating immunogenicity, their presence is a signal of mutational processes that will also have resulted in nonsynonymous mutations and neoantigens elsewhere in the genome.

The form of genomic instability associated with defective DNA mismatch repair in tumors is to be called microsatellite instability (MSI). An algorithm for the detection of somatic microsatellite changes using paired tumor-normal sequence data was applied to all tumors, yielding a quantitative score by MSIsensor (11). Tumors deemed to have inadequate tumor content or quality (<200 × median exon coverage, <10% median exonic variant allele frequency, or no mutations with ≤20% tumor content on pathologic review) were flagged, and their MSIsensor scores were excluded from the primary analysis.

### Cell culture, transfection and CCK-8 assay

We got the ESCC cell lines (KYSE-150 and TE-1) from the cell bank of the Chinese Academy of Sciences (Shanghai, China). Cells were cultured in RPMI 1640 (Hyclone, United States) supplemented with 10% fetal bovine serum (FBS, Gibco, United States) and 1% penicillin/streptomycin under a 5% CO_2_ atmosphere at 37°C. The lentiviruses containing the cDNA encoding NOTCH1 or NOTCH1 shRNA were purchased from Genechem (Shanghai, China) and used to infect the ESCC cells in the presence of 6 μg/ml polybrene (sigma). Cell Counting Kit-8 (CCK-8, CK04, Dojindo, Japan) was utilized to assess cell viability and proliferation. Briefly, 3×10^3^ cells per well were seeded in 96 well microplates. At indicated time points, 10 µL of CCK-8 solution was added to each well and incubated for 2 hours at 37°C. Then, we measured the absorbance values at 450 nm by using the microplate reader.

### Real-time PCR and LDH release assay

Real-time PCR (qPCR) was performed as described previously (12). The primers for NOTCH1 were listed as follows: Forward: 5’-GAGGCGTGGCAGACTATGC-3’, Reverse: 5’- CTTGTACTCCGTCAGCGTGA-3’. An LDH Cytotoxicity Assay kit for LDH release (catalog no. C0017) was purchased from Beyotime (Shanghai, China) and the experiments were performed according to the manufacturer’s instructions.

### Statistical analysis

Using the cluster Profiler package (13), Gene ontology (GO) and Kyoto Encyclopedia of Genes and Genomes (KEGG) pathway enrichment analyses were performed with significantly genes. Correlation was evaluated by statistical R/Bioconductor packages. The Student’s t-test and Fisher’s exact test were utilized to estimate the significance of differences between groups. The values were represented with the mean ± standard error of the mean (SEM) from at least three independent experiments. The *P* value of 0.05 or less was considered statistically significant.

## Results

### Identification of somatic mutations in ESCC

**Figure 1:**
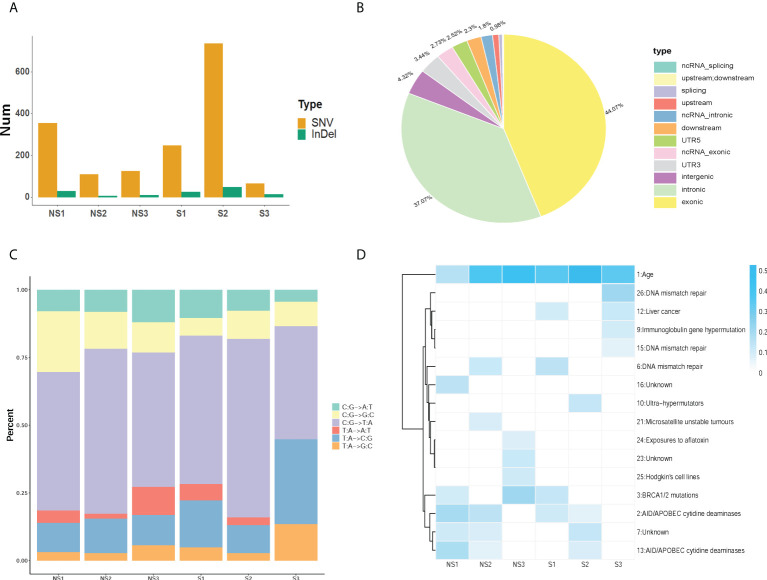
Identification of somatic mutations profiles in ESCC. **(A)** The number of SNVs and InDels was acquired in each sample. **(B)** The statistics of variation location in the genome in ESCC. **(C)** The distribution of somatic mutation type in ESCC patients. **(D)** The mutation signature distribution based on COSMIC signature database. Group S means the radiosensitive group and group NS means the radioresistant group.

According to the response to radiation therapy, ESCC patients were divided into two groups, radio-sensitive and radio-resistant groups. The detailed clinical information was listed in table 1. We performed the whole exome sequencing by utilizing their tumor tissues and paired blood samples. After read quality control, mapping and alignment to the hg19 reference genome, a total of 1,829 somatic variations (median, 209.5; range, 89–807; SD, 271.3) were identified in six patients (**Figure 1A**). The exonic (44.07%) and intronic (37.07%) region accounted for the majority of variation location (**Figure 1B**). The predominant nucleotide changes were C>T and T>C (**Figure 1C**). We analyzed somatic mutation profiles to unravel the mechanism in the radio sensitivity of ESCC (**Figure 1D**). The result indicated that signature Age has high weight in all six ESCC patients. And the insensitive group was enriched in signature AID/APOBEC cytidine deaminases (COSMIC Signatures version 3.2).

### TMB, Intra-tumor Heterogeneity and MSI Comparisons between Radio-resistant and sensitive Groups

It is known to all that there are closely linkages between tumor mutational burdens (TMB) or microsatellite instability (MSI) and the immunotherapy response in the treatment of cancers. Therefore, we analyzed whether TMB, MSI and Intra-tumor Heterogeneity were related with the response to radiotherapy in ESCC. As shown in **Table 2**, the value of TMB ranged from 1.22 to 11.65. Although the correlation coefficient is 0.55, there was no significant difference between the two groups (p=0.26). Moreover, MSI was calculated to estimate the differences as well. A correlation coefficient of 0.29 (p=0.58) between two groups indicates no significant association of MSI with the response to radiotherapy in ESCC patients. To determine the correlation between radiosensitivity and clonality, we further analyzed the purity and copy number alterations in this study. The results showed that the correlation coefficients were -0.02 (P=0.97) and 0.18 (P=0.74), respectively. There was no obvious association between radiosensitivity and clonality in ESCC. In addition, we also studied whether age and tumor stage influenced the response to radiotherapy. The results showed that no significant differences were acquired, indicating that age and tumor stage are not critical factors in the radioresistence of ESCC.

**Table 2:** Difference of TMB, MSI, and purity between radio-sensitive and radio-resistant groups.

Type	NS1	NS2	NS3	S1	S2	S3	Correlation	P.value
**TMB**	5.69	1.74	2.02	4.06	11.65	1.22	0.55	0.26
**MSI (%)**	22.98	28.22	9.3	22.47	24.17	17.13	0.29	0.58
**Purity**	0.39	0.48	0.31	0.79	0.24	0.17	-0.02	0.97
**Ploidy**	2.40	1.66	1.35	1.88	2.17	1.59	0.18	0.74
**Age**	57	77	57	60	67	52	0.10	0.85
**Stage**	3	3	2	3	3	2	0.20	0.70

### Functional analysis of somatic differentially mutated genes

**Figure 2:**
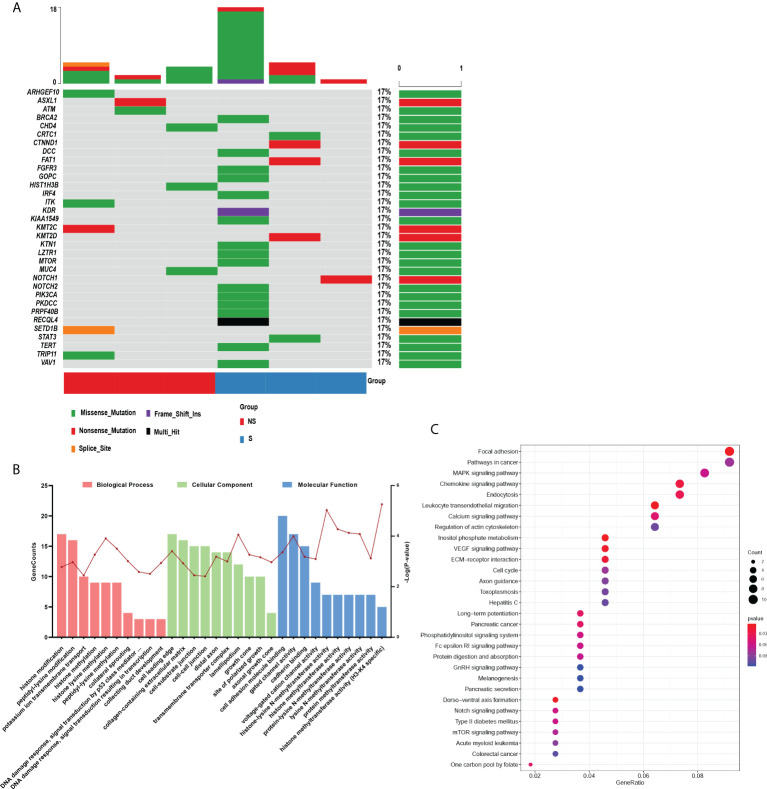
Functional analysis of somatic differentially mutated genes. **(A)** The oncoprint plot of 33 somatic mutations. **(B, C)** GO **(B)** and KEGG **(C)** enrichment analysis were performed on the differential mutant genes. Group S means the radiosensitive group and group NS means the radioresistant group.

According to the criterions mentioned in the methods, 344 differentially mutated genes were filtered from all somatic mutation genes. By referring to the COSMIC cancer gene census, 33 mutated genes were retained and presented in **Figure 2A**. GO and KEGG enrichment analysis were performed on these mutated genes to further explore their functions and effects. As displayed in **Figure 2B**, biological process terms showed that the mutated genes mainly participated in histone modification, transmembrane transport, and several classic signal transduction processes of cancer in response to DNA damage. Additionally, these genes were expressed in cellular components such as cell-cell junction, cell-substrate junction, and collagen−containing extracellular matrix, which was associated with migrations and invasions. Enriched molecular function terms showed the similar results, including cell adhesion molecule binding and methyltransferase activity. The top 30 significantly enriched KEGG pathways mainly included cell cycle, MAPK, VEGF, NOTCH, and mTOR signaling pathways, whose activation or inactivation is closely correlated with cell survival, proliferation and the progression of cancer (**Figure 2C**).

**Figure 3:**
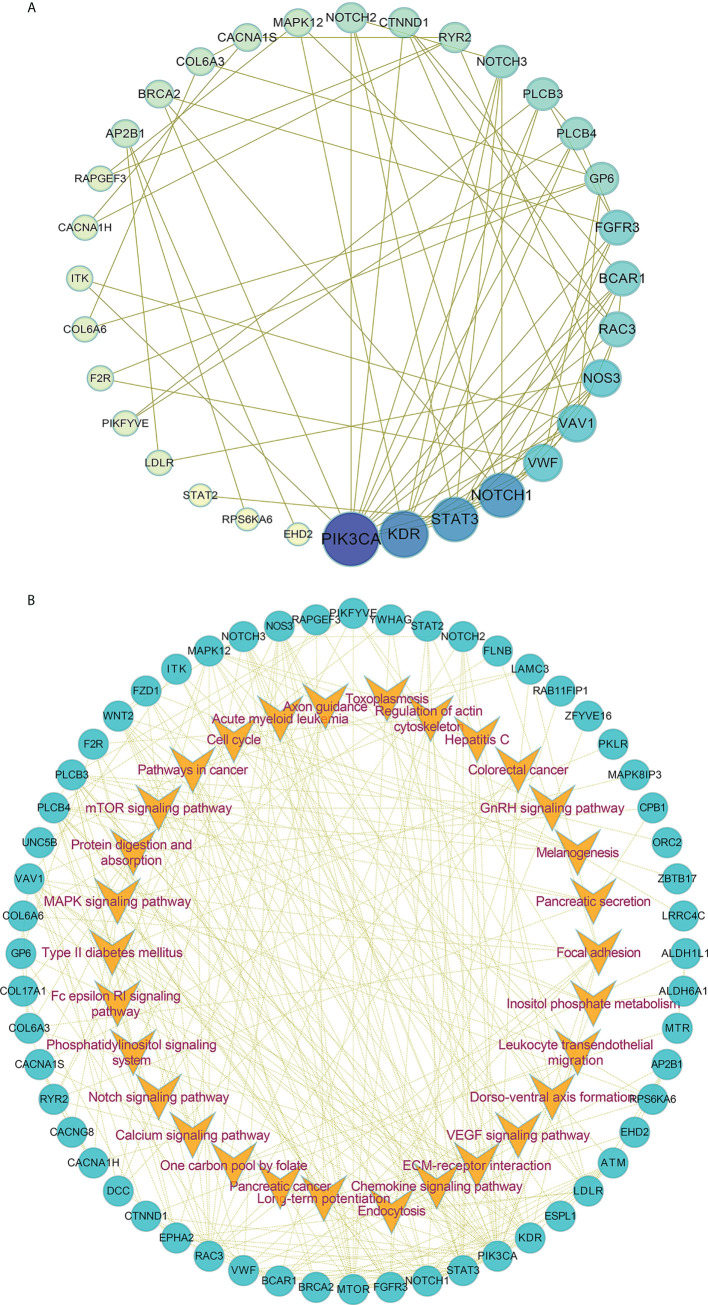
The protein-protein interaction network in ESCC. **(A, B)** The protein-protein interaction network **(A)** by utilizing the differentially mutated genes and pathway-based protein interaction **(B)** were presented.

Furthermore, we also performed the protein-protein interaction (PPI) network by utilizing the differentially mutated genes. As shown in **Figure 3A**, several hub genes with most nodes, such as PIK3CA, NOTCH1, STAT3 and KDR, were identified. Combined with the relative pathways, a pathway-based protein interaction was presented in **Figure 3B**. The mediators and pathways involved in the network were likely related with affecting the radiosensitivity in ESCC.

### Identification of germline mutations in ESCC

**Figure 4:**
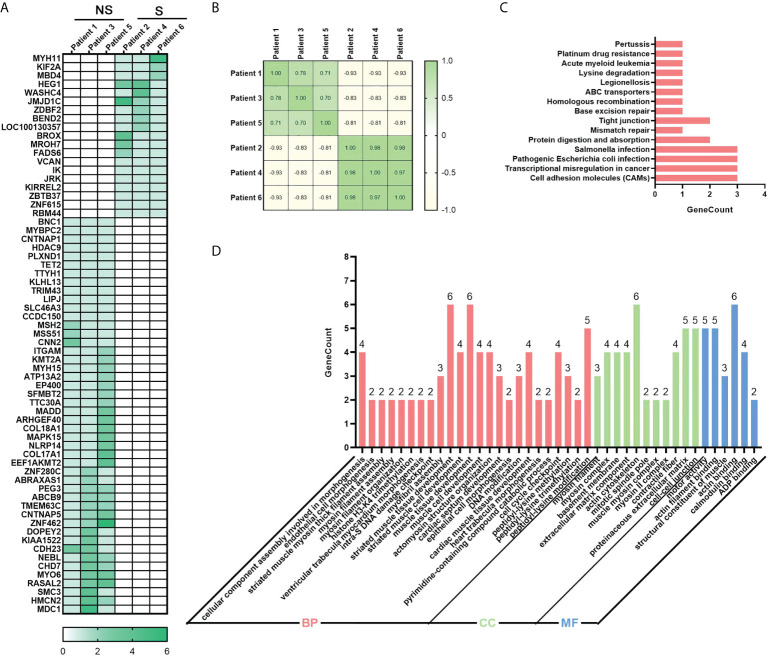
Identification of germline mutations in ESCC. **(A)** Heatmap of 65 differentially mutated genes between radio-sensitive and radio-resistant groups. **(B)** Spearman correlation coefficient analysis of ESCC samples. **(C)** Top 15 enriched KEGG pathway of differentially genes. **(D)** Significantly enriched GO of differentially genes. *P*-value of all GO terms displayed in pictures is smaller than 0.01. Group S means the radiosensitive group and group NS means the radioresistant group.

Meantime, we also examined the genetic differences between the radio-sensitive and radio-resistant groups, 65 germline differentially mutated genes were found. In **Figure 4A**, mutation frequency was shown in blocks of different shade. Compared with the radio-sensitive group, there were more mutated genes and greater mutated rates in the radio-resistant group. After the analysis of differences between groups, spearman correlation coefficients were calculated to evaluate the within-group differences (**Figure 4B**). Positive coefficients (≥0.7) revealed high positive correlation within samples in the same group. To investigate functions of these germline differentially mutated genes, KEGG and GO enrichment analysis were performed. The results highlighted several pathways correlated with DNA damage repair, such as Homologous recombination, Mismatch repair and Base excision repair (**Figure 4C**). GO enrichment analysis showed that the germline differentially mutated genes were predominantly enriched in DNA damage checkpoint, cell cycle checkpoint, basement membrane, extracellular matrix component, and motor activity (**Figure 4D**). The results imply that the germline mutations might be also involved in regulating the radiosensitivity in ESCC.

### NOTCH1 negatively regulates the response to radiotherapy in ESCC

**Figure 5:**
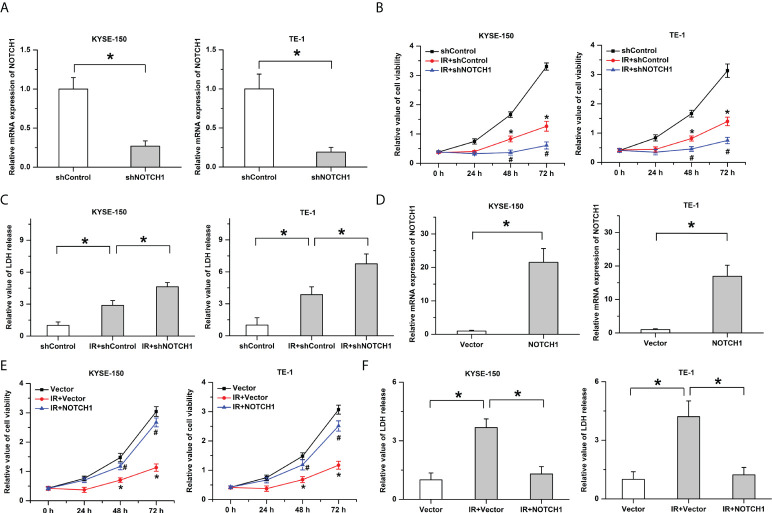
The response to radiotherapy is negatively regulated by NOTCH1. **(A)** The knockdown efficiency was verified by real-time PCR in ESCC cells. **(B)** Ionizing radiation (IR)-induced the decrease of cell viability was facilitated by the knockdown of NOTCH1. * indicates *p* < 0.05 compared with shControl group, # indicates *p* < 0.05 compared with IR+shControl group. **(C)** The promotive effects of IR on LDH release were enhanced by NOTCH1 knockdown. **(D)** Real-time PCR was utilized to determine the overexpression efficiency. **(E)** The decrease of cell viability induced by IR was attenuated by the overexpression of NOTCH1. * indicates *p* < 0.05 compared with Vector group, # indicates *p* < 0.05 compared with IR+Vector group. **(F)** IR-induced cell death was mitigated by NOTCH1 overexpression in ESCC cells. All data was represented as the mean ± standard error of the mean (SEM) from at least three independent experiments. Vector and shControl were utilized as the internal reference to calculated the relative values, respectively.

To determine the role of NOTCH1 (a hub gene of PPI in somatic mutated genes) in the radiosensitivity of ESCC, we then knocked down its expression in ESCC cells (KYSE-150 and TE-1) by shRNA and utilized qPCR to verify the knockdown efficiency (**Figure 5A**). As shown in **Figure 5B**, results of cck-8 showed that treatment with ionizing radiation (IR, 4Gy) led to the decrease of cell viability in ESCC cells. The inhibitory effects of IR on cell growth were enhanced by the knockdown of NOTCH1. Consistently, the knockdown of NOTCH1 facilitated IR-induced cell death as determined by LDH release assay (**Figure 5C**). Furthermore, to demonstrate the physical effects of NOTCH1 on regulating the radiosensitivity of ESCC, we also overexpressed NOTCH1 in ESCC cells (**Figure 5D**). Our result showed that the decrease of cell viability induced by IR was attenuated by the overexpression of NOTCH1 (**Figure 5E**). NOTCH1 overexpression significantly mitigated IR-induced cell death in ESCC cells (**Figure 5F**). These results imply that the response to radiotherapy is negatively regulated by NOTCH1 in ESCC.

### High expression of NOTCH1 was accompanied with the low levels of some immunotherapy-related cells

**Figure 6:**
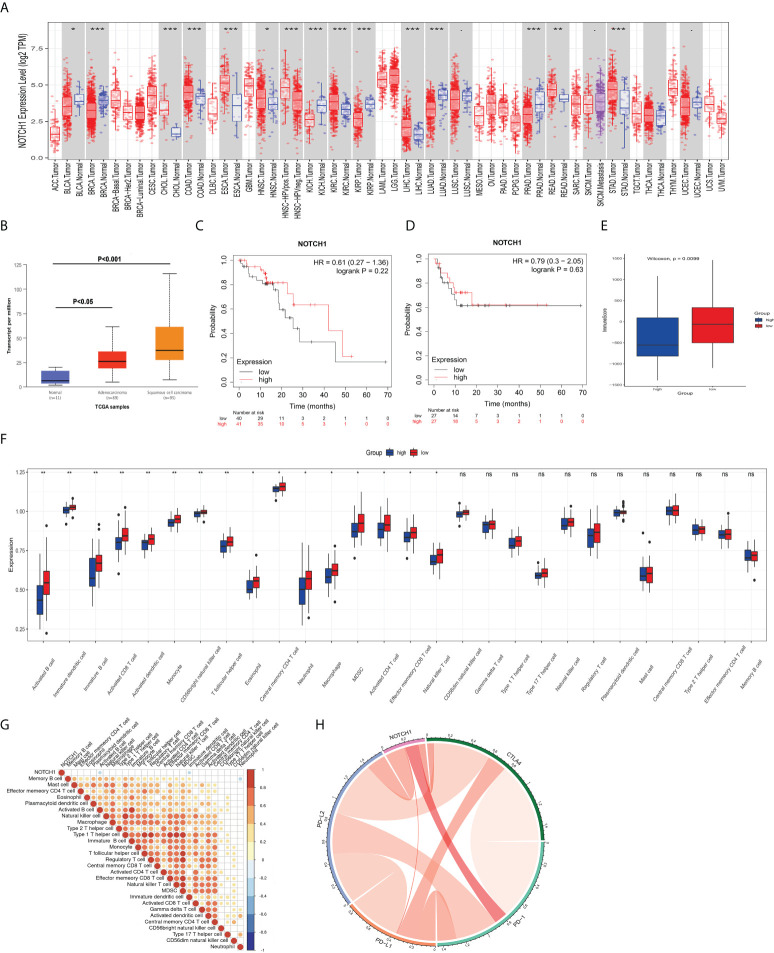
NOTCH1 is correlated with a few immunotherapy-related cells. **(A)** Expression of NOTCH1 in different types of cancers and their normal tissues. **(B)** NOTCH1 was significantly upregulated in esophageal cancer, especially in ESCC. C and D: There were no significant differences on the overall survival **(C)** and recurrence free survival **(D)** between the high levels of NOTCH1 and the low levels of NOTCH1 in ESCC patients. **(E)** The immune score was significantly decreased in the group with high NTOCH1 expression in ESCC. **(F)** Several immune cells, including activated B cell, activated CD8 T cell, activated dendritic cell, and CD56bright natural killer cell, were significantly enriched in the group with low NTOCH1 expression in ESCC. **(G)** Expression of NOTCH1 was negatively correlated with activated B cell and immature dendritic cell. **(H)** There were no significant relations between NOTCH1 and the four immune checkpoint molecules in ESCC. *P<0.05, **P<0.01, ***P<0.001, and ns, P>0.05.

It is widely accepted that the response of tumors to radiotherapy is regulated by multifactorial. Not only intrinsic cellular radioresistance but also tumor immune microenvironment plays important roles in affecting the death of cancer cells after the treatment with radiotherapy (14, 15). To determine whether notch1 also has a regulatory effect on the immune microenvironment in ESCC, we thus analyzed the correlation of notch1 and tumor immune infiltration. As shown in **Figure 6A**, the results showed that the expression of NOTCH1 was not always consistent in different cancers compared with their normal tissues by using the TIMER database. We found that NOTCH1 was frequently up-regulated in some cancers including colon adenocarcinoma, kidney renal clear cell carcinoma and esophageal carcinoma, whereas the expression of NOTCH1 was significantly down-regulated in kidney renal papillary cell carcinoma and lung adenocarcinoma. The analysis results of the UALCAN database showed that the expression of NOTCH1 was significantly increased in esophageal squamous cell carcinoma (**Figure 6B**). To identify the prognosis values of NOTCH1 in ESCC, we then determined whether NOTCH1 was associated with the clinical outcome by utilizing the Kaplan-Meier Plotter (16). As shown in **Figure 6C, D**, there were no significant differences on the overall survival (OS) and recurrence free survival (RFS) between the high levels of NOTCH1 and the low levels of NOTCH1 in ESCC patients. Furthermore, we also examined the differences of immune cell based on the expression of NOTCH1 in ESCC. As shown in **Figure 6E**, the immune score was significantly decreased in the group with high NTOCH1 expression. Moreover, we found several immune cells, including activated B cell, activated CD8 T cell, activated dendritic cell, monocyte and CD56bright natural killer cell, were significantly enriched in the group with low NTOCH1 expression in ESCC (**Figure 6F**). To further explore the potential relationships between NOTCH1 expression and immune cell type, we performed the correlation analyses. Our results showed that the expression of NOTCH1 was negatively correlated with activated B cell (r=-0.2755, P=0.0175) and immature dendritic cell (r=-0.2409, P=0.0387) (**Figure 6G**). In addition, it is widely accepted that checkpoint inhibitors play important roles in the therapy of cancer. We then examined the relationship between NOTCH1 and the four immune checkpoint molecules. However, no significant relations were observed between NOTCH1 and the four immune checkpoint molecules in ESCC (**Figure 6H**). The results indicate that activated B cell and immature dendritic cell likely participate in NOTCH1-regulated the sensitivity of radiotherapy in ESCC.

## Discussion

It is widely known that radiation therapy is an important strategy for the treatment of cancers, especially in ESCC. However, due to the existence of inherent or acquired radiation resistance, the effect of radiotherapy is far from meeting people’s expectations. Many patients with ESCC have not been significantly improved after radiotherapy. Therefore, it is necessary to determine the critical factors involved in affecting the radiosensitivity of ESCC. In the present study, we identified some somatic and germline mutations of genes and constructed the pathway-based protein interaction network by performing the whole-exome sequencing, which were potentially associated with the response to radiotherapy in ESCC. Moreover, *in vitro* studies showed that the inhibitory effects of IR on cell survival were negatively regulated by NOTCH1 in ESCC cells. The results provide some potentially new treatment targets for improving the sensitivity of radiation in ESCC.

One of the most important findings in this study is that some somatic mutated genes potentially associated with the radiosensitivity were identified in ESCC. The hub genes (such as STAT3, PIK3CA and NOTCH1) in protein-protein interaction network likely play important roles in the response to radiotherapy. STAT3 as a signal transducer and transcription activator mediates many cellular physiological processes, including cell proliferation, survival, angiogenesis and inflammatory response. Accumulating evidence has implicated that Stat3 plays an important role in regulating the response to radiotherapy in ESCC. Previous studies have shown that inhibition of the STAT3 signaling axis in ESCC cells increases radiosensitivity by inducing apoptosis and enhancing DNA damage after radiotherapy (17, 18). Besides, the epithelial–mesenchymal transition induced by ionizing radiation and radioresistance are attenuated by STAT3 inhibition in ESCC (19). The conclusions are in accordance with our result that mutation of STAT3 was acquired in the radiosensitive group in ESCC. PIK3CA activates AKT1 by the stimulations of receptor tyrosine kinase ligands such as EGF, insulin, IGF1, VEGFA and PDGF, leading to activating signaling cascades involved in cell growth, survival, proliferation, motility and morphology (20). It is reported that hyper-activation of PI3K is frequently observed in ESCC tissues and selective targeting PI3Kα has been considered as a promising strategy for the ESCC therapy (21). DNA damage, G2/M arrest and apoptosis induced by radiotherapy are facilitated by PI3Kα inhibition in ESCC, the sensitivity to radiation is increased by PI3Kα inhibitors in esophageal squamous cell carcinoma (22). PIK3CA mutation is associated with a better disease-free survival and overall survival in esophageal squamous cell carcinoma (23). It is in accordance with our study that mutation of PIK3CA was acquired in the radiosensitive group. NOTCH1 functions as a receptor for membrane-bound ligands Jagged-1 (JAG1), Jagged-2 (JAG2) and Delta-1 (DLL1) to regulate cell-fate determination and deregulation of the Notch pathway participates in regulating the initiation and progression of tumors (24, 25). It is reported that inhibition of the notch1 transcriptional complex suppresses tumor growth by targeting cancer stem cells in ESCC (24). NOTCH1-induced stemness promotes the resistance to chemotherapy or radiotherapy in head and neck squamous cell carcinomas cells (26). Our results indicated that mutation of NOTCH1 was observed in the radiosensitive group in ESCC, which was consistent with a previous study (27). Moreover, we found that the knockdown of NOTCH1 facilitated the inhibitory effects of IR on the growth of ESCC cells, whereas IR-induced cell death was attenuated by the overexpression of NOTCH1. The results indicate that NOTCH1 acts as a negative regulator of radiosensitivity in ESCC. However, further studies are still needed to determine the regulatory mechanisms of the somatic mutated genes related with affecting the radiosensitivity in ESCC.

Accumulating evidence has shown that DNA damage repair has a clear role in resistance of anti-cancer radio-/chemo-therapies (28). Irradiation with sensitizer can cause potential chemical lethal damage to cells, such as single-strand breaks (SSBs), chemically altered base lesions, abasic sites, interstrand crosslinks, intrastrand crosslinks, and most consequentially, double-strand breaks (DSBs) (29). Six major pathways for DNA repair, including direct reversal, base excision repair, mismatch repair, nucleotide excision repair, homologous recombination and non-homologous end-joining, have been identified (30). Congenital DNA repair deficiency can cause the accumulation of DNA damages leading to cell death or malignant transformation into tumor cells. In our study, we found that several pathways correlated with DNA damage repair, including homologous recombination, mismatch repair and base excision repair, were addressed in the results of analyzing germline mutations. Genes (such as ABRAXAS1 and MBD4) had significantly different variant status between the radio-resistant and the radio-sensitive groups. It is reported that heterozygous germline mutations in ABRAXAS1 plays important roles in mitigating DNA damage response and inhibiting deregulated G2-M checkpoint control (31). SNP in coding regions of MBD4 Glu346Lys has been identified as a significant predictor for the risk of ESCC (32). Deficiency of MBD4 inhibits the normal apoptotic response to gamma-irradiation and DNA-damaging agents (33). Our results showed that mutations of ABRAXAS1 and MBD4 were frequently observed in the radio-resistant and the radio-sensitive groups, respectively. The findings indicate that these germline mutations likely also participate in regulating the response to radiotherapy in ESCC.

Accumulating evidence has indicated that tumor immune microenvironment plays important roles in affecting the death of cancer cells (14). Several biological responses, DNA damage repair and the changes in tumor inflammatory microenvironments, are involved in the death of cancer cell induced by radiotherapy (15). Radiotherapy, in addition to direct cytotoxic effect on tumor cells, could reprogram the immune microenvironment of tumors by regulating the release of inflammatory mediators and the infiltrating immunostimulatory cells (34). Antitumor adaptive immunity could be evoked by the treatment with radiation therapy (35). Moreover, radiotherapy in combination with immunotherapy has been performed in some clinical trials of cancers. The inhibitory effects of radiotherapy on the growth, recurrence and metastasis of cancers were significantly ameliorated by the blockade of immune checkpoints in the experimental study and clinical observations (36, 37). Previous studies have shown that Notch1 represses the infiltration of CD8(+) cytotoxic T lymphocytes and NK cells and inhibits the release of IFN-γ in melanoma (38). Notch1 positively regulates the immune suppressive cells and inhibits the recruitment of functional CD8(+) T cells. Inhibition of NOTCH1 facilitated the efficacy of immunotherapy in melanoma (39). It is reported that Notch1 is correlated with immune infiltrates in gastric cancer (40). Deleterious NOTCH Mutation leads to the increased transcription of genes related to DNA damage response and immune activation in NSCLC (41). Until now, the effects of NOTCH1 on tumor immune microenvironment in ESCC remain largely unknown. In the present study, we further examined the relationship between NOTCH1 and immune cells type in ESCC. The results showed that some immune cells, such as activated CD8 T cell, activated dendritic cell, and CD56bright natural killer cell, were significantly decreased in the group with high NTOCH1 expression in ESCC. Moreover, activated B cell and immature dendritic cell were negatively correlated with the expression of NOTCH1. However, there were no significant relations between NOTCH1 and the four immune checkpoint molecules. Although the results imply that NTOCH1 likely participate in regulating the tumor immune microenvironment in ESCC, its specific physiological roles and the corresponding regulatory mechanisms were still needed to be elaborated in further studies. Meanwhile, there are some limitations in the research, which needs to be addressed in future experiments. The sample size utilized for bioinformatics data analysis was small and the roles of NOTCH1 in the response to radiotherapy were just validated by *in vitro* study. We will further collect the ESCC samples to determine the effects of the key genes (especially NOTCH1) on radiosensitivity and tumor immune infiltration and perform *in vivo* experiments to demonstrate the regulatory mechanisms of NOTCH1 in ESCC.

## Conclusions

In summary, the present study examined the differences of the germline mutations and somatic mutations between the radiosensitive and radioresistence groups in ESCC. We also identified the critical mutations of candidate genes, which were likely associated with the response to radiotherapy. The findings might provide some potential biomarkers and candidate targets for improving the efficiency of radiotherapy in ESCC.

## Data availability statement

The original contributions presented in the study are publicly available. This data can be found here: https://www.ncbi.nlm.nih.gov/sra Accession number is PRJNA870670

## Ethics statement

The studies involving human participants were reviewed and approved by Renji Hospital. The patients/participants provided their written informed consent to participate in this study. Written informed consent was obtained from the individual(s) for the publication of any potentially identifiable images or data included in this article.

## Author contributions

LZ, JM, and XM designed this study. LZ, XX, YW, JL, YG and YB analyzed the data. LZ and JM wrote the manuscript and did the experiments. XX, YW, XW, LR and JT collected the data. All authors contributed to the article and approved the submitted version.

## Funding

This study was supported by National Natural Science Foundation of China (81972854), Science and Technology Commission of Shanghai Municipality (21ZR1438500), Renji Hospoital promotion project of National Natural Science Foundation of China (RJTJ22-MS-030), the Incubating Program for Clinical Innovation of Renji Hospital (PYDY-DZX-009) and the Beijing Xisike Clinical Oncology Research Foundation (Y-XD202001/zb-0011).

## Conflict of interest

The authors declare that the research was conducted in the absence of any commercial or financial relationships that could be construed as a potential conflict of interest.

The reviewer ZW declared a shared parent affiliation with the authors XX, YW, YB, XW, LR, JT, XM, LZ to the handling editor at the time of the review.

## Publisher’s note

All claims expressed in this article are solely those of the authors and do not necessarily represent those of their affiliated organizations, or those of the publisher, the editors and the reviewers. Any product that may be evaluated in this article, or claim that may be made by its manufacturer, is not guaranteed or endorsed by the publisher.

## References

[1] Bray F Ferlay J Soerjomataram I Siegel RL Torre LA Jemal A. Global cancer statistics 2018: GLOBOCAN estimates of incidence and mortality worldwide for 36 cancers in 185 countries. CA Cancer J Clin (2018) 68:394–424. doi: 10.3322/caac.21492 30207593

[2] Holmes RS Vaughan TL. Epidemiology and pathogenesis of esophageal cancer. Semin Radiat Oncol (2007) 17:2–9. doi: 10.1016/j.semradonc.2006.09.003 17185192

[3] Sun J Huang W Chen J Zhang Y. Association of 3D-CRT and IMRT accelerated hyperfractionated radiotherapy with local control rate and 5-year survival in esophageal squamous cell carcinoma patients. Br J Radiol (2022) 95: 20211195. doi: 10.1259/bjr.20211195 35119916 PMC10993959

[4] Chen GZ Zhu HC Dai WS Zeng XN Luo JH Sun XC. The mechanisms of radioresistance in esophageal squamous cell carcinoma and current strategies in radiosensitivity. J Thorac Dis (2017) 9:849–59. doi: 10.21037/jtd.2017.03.23 PMC5394057 28449496

[5] Sun Y Wang J Ma Y Li J Sun X Zhao X. Radiation induces NORAD expression to promote ESCC radiotherapy resistance *via* EEPD1/ATR/Chk1 signalling and by inhibiting pri-miR-199a1 processing and the exosomal transfer of miR-199a-5p. J Exp Clin Cancer Res (2021) 40:306. doi: 10.1186/s13046-021-02084-5 34587992 PMC8479908

[6] Zheng ZY Yang PL Luo W Yu SX Xu HY Huang Y. STAT3β enhances sensitivity to concurrent chemoradiotherapy by inducing cellular necroptosis in esophageal squamous cell carcinoma. Cancers (Basel) (2021) 13 :901. doi: 10.3390/cancers13040901 33670049 PMC7926856

[7] Xu P Zhang Y Ge F Zhang F He X Gao X. Modulation of tumor microenvironment to enhance radiotherapy efficacy in esophageal squamous cell carcinoma by inhibiting carbonic anhydrase IX. Front Oncol (2021) 11:637252. doi: 10.3389/fonc.2021.637252 34249682 PMC8267588

[8] Chen S Zhou Y Chen Y Gu J. Fastp: An ultra-fast all-in-one FASTQ preprocessor. Bioinformatics (2018) 34:i884–90. doi: 10.1093/bioinformatics/bty560 PMC6129281 30423086

[9] Li H Handsaker B Wysoker A Fennell T Ruan J Homer N. The sequence Alignment/Map format and SAMtools. Bioinformatics (2009) 25:2078–9. doi: 10.1093/bioinformatics/btp352 PMC2723002 19505943

[10] McKenna A Hanna M Banks E Sivachenko A Cibulskis K Kernytsky A. The genome analysis toolkit: A MapReduce framework for analyzing next-generation DNA sequencing data. Genome Res (2010) 20:1297–303. doi: 10.1101/gr.107524.110 PMC2928508 20644199

[11] Niu B Ye K Zhang Q Lu C Xie M McLellan MD. MSIsensor: microsatellite instability detection using paired tumor-normal sequence data. Bioinformatics (2014) 30:1015–6. doi: 10.1093/bioinformatics/btt755 PMC3967115 24371154

[12] Hu B Ma X Huang R Wu Z Lu J Guo Y. Identification of key genes mutations associated with the radiosensitivity by whole exome sequencing in pancreatic cancer. Front Oncol (2021) 11:697308. doi: 10.3389/fonc.2021.697308 34434896 PMC8381198

[13] Yu G Wang LG Han Y He QY. clusterProfiler: An r package for comparing biological themes among gene clusters. Omics (2012) 16:284–7. doi: 10.1089/omi.2011.0118 PMC3339379 22455463

[14] Willers H Azzoli CG Santivasi WL Xia F. Basic mechanisms of therapeutic resistance to radiation and chemotherapy in lung cancer. Cancer J (2013) 19:200–7. doi: 10.1097/PPO.0b013e318292e4e3 PMC3668666 23708066

[15] Dillon MT Bergerhoff KF Pedersen M Whittock H Crespo-Rodriguez E Patin EC. ATR inhibition potentiates the radiation-induced inflammatory tumor microenvironment. Clin Cancer Res (2019) 25:3392–403. doi: 10.1158/1078-0432.CCR-18-1821 PMC6551222 30770349

[16] Nagy Á. Munkácsy G Győrffy B. Pancancer survival analysis of cancer hallmark genes. Sci Rep (2021) 11:6047. doi: 10.1038/s41598-021-84787-5 33723286 PMC7961001

[17] Sugase T Takahashi T Serada S Fujimoto M Hiramatsu K Ohkawara T. SOCS1 gene therapy improves radiosensitivity and enhances irradiation-induced DNA damage in esophageal squamous cell carcinoma. Cancer Res (2017) 77:6975–86. doi: 10.1158/0008-5472.CAN-17-1525 29042418

[18] Yu D Ma Y Feng C Ma Z Guo J Chen H. PBX1 increases the radiosensitivity of oesophageal squamous cancer by targeting of STAT3. Pathol Oncol Res (2020) 26:2161–8. doi: 10.1007/s12253-020-00803-5 32170580

[19] Zang C Liu X Li B He Y Jing S He Y. IL-6/STAT3/TWIST inhibition reverses ionizing radiation-induced EMT and radioresistance in esophageal squamous carcinoma. Oncotarget (2017) 8:11228–38. doi: 10.18632/oncotarget.14495 PMC5355260 28061440

[20] Yamaguchi H Yoshida S Muroi E Yoshida N Kawamura M Kouchi Z. Phosphoinositide 3-kinase signaling pathway mediated by p110α regulates invadopodia formation. J Cell Biol (2011) 193:1275–88. doi: 10.1083/jcb.201009126 PMC3216328 21708979

[21] Elkabets M Pazarentzos E Juric D Sheng Q Pelossof RA Brook S. AXL mediates resistance to PI3Kα inhibition by activating the EGFR/PKC/mTOR axis in head and neck and esophageal squamous cell carcinomas. Cancer Cell (2015) 27:533–46. doi: 10.1016/j.ccell.2015.03.010 PMC4398915 25873175

[22] Shi JJ Xing H Wang YX Zhang X Zhan QM Geng MY. PI3Kα inhibitors sensitize esophageal squamous cell carcinoma to radiation by abrogating survival signals in tumor cells and tumor microenvironment. Cancer Lett (2019) 459:145–55. doi: 10.1016/j.canlet.2019.05.040 31173854

[23] Shigaki H Baba Y Watanabe M Murata A Ishimoto T Iwatsuki M. PIK3CA mutation is associated with a favorable prognosis among patients with curatively resected esophageal squamous cell carcinoma. Clin Cancer Res (2013) 19:2451–9. doi: 10.1158/1078-0432.CCR-12-3559 23532889

[24] Alvarez-Trotta A Guerrant W Astudillo L Lahiry M Diluvio G Shersher E. Pharmacological disruption of the Notch1 transcriptional complex inhibits tumor growth by selectively targeting cancer stem cells. Cancer Res (2021) 81:3347–57. doi: 10.1158/0008-5472.CAN-20-3611 PMC8655881 33820800

[25] Brütsch R Liebler SS Wüstehube J Bartol A Herberich SE Adam MG. Integrin cytoplasmic domain-associated protein-1 attenuates sprouting angiogenesis. Circ Res (2010) 107:592–601. doi: 10.1161/CIRCRESAHA.110.217257 20616313

[26] Byun JY Huang K Lee JS Huang W Hu L Zheng X. Targeting HIF-1α/NOTCH1 pathway eliminates CD44(+) cancer stem-like cell phenotypes, malignancy, and resistance to therapy in head and neck squamous cell carcinoma. Oncogene (2022) 41 :1352–63. doi: 10.1038/s41388-021-02166-w 35013621

[27] Yang L Zhang X MacKay M Foox J Hou Q Zheng X. Identification of radioresponsive genes in esophageal cancer from longitudinal and single cell exome sequencing. Int J Radiat Oncol Biol Phys (2020) 108:1103–14. doi: 10.1016/j.ijrobp.2020.06.015 32561500

[28] Weichselbaum RR Ishwaran H Yoon T Nuyten DS Baker SW Khodarev N. An interferon-related gene signature for DNA damage resistance is a predictive marker for chemotherapy and radiation for breast cancer. Proc Natl Acad Sci U S A (2008) 105:18490–5. doi: 10.1073/pnas.0809242105 PMC2587578 19001271

[29] Blaisdell JO Harrison L Wallace SS. Base excision repair processing of radiation-induced clustered DNA lesions. Radiat Prot Dosimetry (2001) 97:25–31. doi: 10.1093/oxfordjournals.rpd.a006634 11763354

[30] Nordstrand LM Ringvoll J Larsen E Klungland A. Genome instability and DNA damage accumulation in gene-targeted mice. Neuroscience (2007) 145:1309–17. doi: 10.1016/j.neuroscience.2006.10.059 17218062

[31] Bose M Sachsenweger J Laurila N Parplys AC Willmann J Jungwirth J. BRCA1 mislocalization leads to aberrant DNA damage response in heterozygous ABRAXAS1 mutation carrier cells. Hum Mol Genet (2019) 28:4148–60. doi: 10.1093/hmg/ddz252 31630195

[32] Hao B Wang H Zhou K Li Y Chen X Zhou G. Identification of genetic variants in base excision repair pathway and their associations with risk of esophageal squamous cell carcinoma. Cancer Res (2004) 64:4378–84. doi: 10.1158/0008-5472.CAN-04-0372 15205355

[33] Sansom OJ Zabkiewicz J Bishop SM Guy J Bird A Clarke AR. MBD4 deficiency reduces the apoptotic response to DNA-damaging agents in the murine small intestine. Oncogene (2003) 22:7130–6. doi: 10.1038/sj.onc.1206850 14562041

[34] Liauw SL Connell PP Weichselbaum RR. New paradigms and future challenges in radiation oncology: An update of biological targets and technology. Sci Transl Med (2013) 5:173sr2. doi: 10.1126/scitranslmed.3005148 23427246 PMC3769139

[35] Lee Y Auh SL Wang Y Burnette B Wang Y Meng Y. Therapeutic effects of ablative radiation on local tumor require CD8+ T cells: changing strategies for cancer treatment. Blood (2009) 114:589–95. doi: 10.1182/blood-2009-02-206870 PMC2713472 19349616

[36] Deng L Liang H Burnette B Beckett M Darga T Weichselbaum RR. Irradiation and anti-PD-L1 treatment synergistically promote antitumor immunity in mice. J Clin Invest (2014) 124:687–95. doi: 10.1172/JCI67313 PMC3904601 24382348

[37] Postow MA Callahan MK Barker CA Yamada Y Yuan J Kitano S. Immunologic correlates of the abscopal effect in a patient with melanoma. N Engl J Med (2012) 366:925–31. doi: 10.1056/NEJMoa1112824 PMC3345206 22397654

[38] Yang Z Qi Y Lai N Zhang J Chen Z Liu M. Notch1 signaling in melanoma cells promoted tumor-induced immunosuppression *via* upregulation of TGF-β1. J Exp Clin Cancer Res (2018) 37:1. doi: 10.1186/s13046-017-0664-4 29301578 PMC5755139

[39] Qiu H Zmina PM Huang AY Askew D Bedogni B. Inhibiting Notch1 enhances immunotherapy efficacy in melanoma by preventing Notch1 dependent immune suppressive properties. Cancer Lett (2018) 434:144–51. doi: 10.1016/j.canlet.2018.07.024 PMC7185871 30036609

[40] Hu J Yu J Gan J Song N Shi L Liu J. Notch1/2/3/4 are prognostic biomarker and correlated with immune infiltrates in gastric cancer. Aging (2020) 12:2595–609. doi: 10.18632/aging.102764 PMC7041744 32028262

[41] Zhang K Hong X Song Z Xu Y Li C Wang G. Identification of deleterious NOTCH mutation as novel predictor to efficacious immunotherapy in NSCLC. Clin Cancer Res (2020) 26:3649–61. doi: 10.1158/1078-0432.CCR-19-3976 32241817

